# Editorial: Enzyme Biocatalysts: Design and Application

**DOI:** 10.3389/fchem.2022.851857

**Published:** 2022-02-02

**Authors:** Jiandong Cui, Gao-Wei Zheng, Gary Black, Hafiz M. N. Iqbal, Muhammad Bilal

**Affiliations:** ^1^ State Key Laboratory of Food Nutrition and Safety, Laboratory of Industrial Fermentation Microbiology, Ministry of Education, Tianjin University of Science and Technology, Tianjin Economic and Technological Development Area (TEDA), Tianjin, China; ^2^ East China University of Science and Technology Shanghai, Shanghai, China; ^3^ Applied Sciences, Northumbria University, Newcastle upon Tyne, United Kingdom; ^4^ Tecnologico de Monterrey, School of Engineering and Sciences, Monterrey, Mexico; ^5^ School of Life Science and Food Engineering, Huaiyin Institute of Technology, Huaian, China

**Keywords:** biocatalysis, enzyme engineering, immobilization, thermal stability, rare sugar, bioinformatic analysis, industrial biotechnology

With the looming concern of climate change, extensive environmental deterioration and mass extinctions, the transition to a greener, environmentally friendly, and sustainable production of liquid fuels and platform chemicals has become imperative. Thanks to the current advancements in bioinformatics, genomics, and protein engineering approach, catalysis with enzymes, i.e., biocatalysis, has emerged as the most powerful technology for widespread industrial applications by minimizing waste, reducing energy necessities, and increasing catalytic reactions (Sheldon et al., 2020; Wu et al., 2021). Enzymes are highly efficient, biocompatible, and biodegradable catalysts and are produced from bio-renewable resources. In contrast to chemical catalysts, enzymatic reactions are carried out at nearly ambient pressures and temperatures at physiological pH, leading to high reaction rates and selectivity. Moreover, protection or activation of functional groups is not required, and enzyme-mediated biocatalytic processes are atom and step economic, resulting in minimal waste and energy consumption than traditional counterparts. To sum up, biocatalytic processes are more economical, sustainable and possess a lower environmental footprint than conventional processes for manufacturing commodity chemicals (Sheldon et al., 2021).

Enzymes have gained increasing importance for producing valuable chemicals, pharmaceuticals, agrochemicals, fuels, and other commodity bio-products in industrial processes. Nevertheless, the fragile nature of native enzymes prevents their utilization under challenging environments, where the industrial bioprocesses generally occur, causing low activity and inadequate stability. Several approaches, including enzyme immobilization, chemical modifications, integrated chemo- and biocatalysis, and designing (micro)environments for biocatalysts, have become the rapidly expanding areas of research to develop industrial biocatalysts with high stabilities, exquisite selectivities, and special activities with non-natural substrates in the unfavourable milieu of high temperatures and elevated substrate concentrations (Ren et al., 2019). In the last 2 decades, biocatalysis has witnessed immense progress and phenomenal growth for transforming into a mature technology with a vast industrial perspective. This development was mainly attributed to the advances in genomics, biochemistry, bioinformatics, synthetic biology, protein engineering, and other computational tools (Bilal et al., 2019).

In total, seven articles were published in this Research Topic summarizing various aspects of enzymes like identification, characterization, improving catalytic attributes and applications in multiple sectors. These were not explicitly focused on a specific domain and relatively more comprehensively, connecting new enzymes’ exploration with biocatalytic applications. For example, Wang et al. identified a potential putative D-Tagatose 3-Epimerase DTEase in the genome of *Christensenella minuta* DSM 22607 (DTE-CM). This newly recognised DTE-CM was utilized for the biocatalytic preparation of an important rare sugar, D-allulose. For the first time, Zhu et al. identified and characterized an O-Succinyl-L-Homoserine Sulfhydrylase from Thioalkalivibrio sulfidiphilus, showing an appreciable substrate tolerance, higher conversion efficiency, and high yield of L-methionine, indicating its applicability for large-scale L-methionine biosynthesis. Zhang et al. explored the relevance of cytochrome P450 CYP109B1 for stereo- and regioselective steroid hydroxylation by screening redox pairs from various resources or creating the fused enzymes by integrating CYP109B1 to the N-terminal of reductase domain of P450RhF and P450 BM3. The CYP109B1 presented outstanding selectivity and catalytic performance for four testosterone derivatives, giving rise to all 15β-hydroxylated steroids as the major products. In another work, the potential mutation sites were substituted with electrically neutral amino acids on the surface of protein for constructing the derivatives with improved catalytic activity and thermal stability of transaminase from *Aspergillus terreus* (Cao et al.). Liang et al. developed and optimized the T7-like and T7 expression system in *Pseudomonas putida* KT2440 to provide a set of corresponding plasmids and related chassis to enhance the recombinant expression level difficult-to-express proteins.

Recently, application of sulfoxide reductase to prepare chiral sulfoxides via kinetic resolution has appeared as an attractive strategy with intriguing catalytic features. Peng et al. discussed the biological and chemical functions of sulfoxide reductases and highlighted their prospects in the biocatalytic preparation of chiral sulfoxide. Wang et al. focused on current research advancement on non-active sites of enzymes. They described two primary research methods with non-catalytic regions as direct targets, including enzyme engineering and interpreting enzymatic mechanisms. In addition to molecular mechanisms and classifying the positions of non-active sites in enzyme structures, bioinformatic analysis of mining non-active sites as targets for protein engineering was also outlined. Altogether, all articles in this Research Topic demonstrate the importance of enzymes in a variety of biotechnological applications.
